# Antifungal-resistant Mucorales in different indoor environments

**DOI:** 10.1080/21501203.2018.1551251

**Published:** 2018-11-26

**Authors:** Liliana Aranha Caetano, Tiago Faria, Jan Springer, Juergen Loeffler, Carla Viegas

**Affiliations:** a H&TRC- Health & Technology Research Center, ESTeSL- Escola Superior de Tecnologia da Saúde, Instituto Politécnico de Lisboa, Lisbon, Portugal; b Research Institute for Medicines (iMed.ULisboa), Faculty of Pharmacy, University of Lisbon, Lisbon, Portugal; c Centro de Ciências e Tecnologias Nucleares, Instituto Superior Técnico, Universidade de Lisboa, Lisbon, Portugal; d Medizinische Klinik und Poliklinik II, University Hospital Wuerzburg, Wuerzburg, Germany; e Centro de Investigação em Saúde Pública, Escola Nacional de Saúde Pública, Universidade NOVA de Lisboa, Lisbon, Portugal

**Keywords:** Mucorales, antifungal drug-resistance, indoor environments

## Abstract

This paper sought to address the prevalence of Mucorales in different indoor environments in Portugal. Environmental samples (183 in total) were collected at dwellings (*n* = 79) and workplaces (bakeries, swine farms, taxis, waste-sorting plants) (*n* = 93) by passive sampling using electrostatic dust collector (EDC), air-conditioning filters, litter, and/or raw materials. Samples were inoculated onto non-selective MEA and DG18 media and were screened for antifungal drug-resistance in azole-supplemented agar Sabouraud media. A probe-based Mucorales-specific real-time PCR assay (Muc18S) was used to detect Mucorales in complement to conventional culture-based methods. Mucorales order was found as more prevalent in air-conditioning filters from waste-sorting fork lifters (35.7%). Amongst Mucorales isolates able to grow in azole-supplemented media, 16 isolates of *Mucor* sp., *Rhizopus* sp. or *Rhizomucor* sp. were not susceptible to 1 mg/L voriconazole, and four isolates of *Mucor* sp. or *Rhizopus* sp. were not susceptible to 4 mg/L itraconazole. In conclusion, combination of the culture-based and molecular methods proved to be reliable for Mucorales order identification in complex environmental samples.

## Introduction

The Mucorales order represents a broad and heterogeneous taxon, being amongst the most ancient groups within the fungal kingdom. In Europe, the most commonly identified human pathogenic genera are *Rhizopus* and *Lichtheimia* (synonyms: *Absidia* or *Mycocladus*), followed by *Mucor, Rhizomucor* and *Cunninghamella* (Kwon-Chung 2012; Lanternier et al. 2012). Mucorales order includes a large number of ubiquitous saprophytes species that can cause severe infections, such as mucormycosis (previously described as zygomycosis).

Mucormycosis is associated with a great deal of morbidity, especially amongst immunocompromised individuals and/or individuals with granulocytopenia and uncontrolled diabetes mellitus. Individuals with recognised primary and secondary immunodeficiency disorders are at an increased risk of infection by a wide range of opportunistic fungi; the risk of infection varies with the degree and nature of the specific immunodeficiency. The frequency and relative importance of these infections have become common in industrialised countries, including Portugal (Sabino et al. 2017), likely due to the increasing number of immunocompromised individuals in the last decades.

The prevalence of mucormycosis worldwide and in Portugal is poorly known. Although improved diagnosis and antifungal prophylaxis in clinical practice have contributed to decrease the incidence of fungal diseases caused by *Candida* and *Aspergillus*, infections by *Fusarium* and Mucorales are on the rise (Kontoyiannis et al. 2005; Bitar et al. 2009; Auberger et al. 2012). Some studies report the incidence of invasive mucormycosis up to 13% in high-risk patients (Petrikkos et al. 2012). However, the incidence of invasive mucormycosis might be underestimated, as mucormycosis can be frequently misdiagnosed as aspergillosis or other fungal invasive diseases, due to biased clinical manifestations and non-specific standard culture-based diagnostic tests used for the detection of fungal infections (Lackner et al. 2014).

Antifungal drug-resistance has been reported for invasive fungal infections caused by *Candida* sp. and *Aspergillus* sp. (Cuenca-Estrella 2014), and for mucormycosis, showing that Mucorales are not susceptible to voriconazole (Caramalho et al. 2017). One concern for Mucorales, as for other fungi, is the emergence of azole resistant strains in the environment that display cross-resistance to clinical azoles, posing unforeseen clinical challenges in the management of severe fungal infections (Leathers and Sypherd 1985; Cuenca-Estrella 2014). The emergence of antifungal-resistant microbes in the clinical and in the environment is an inevitable drawback of exposure to antifungal drugs or related substances that potentially leads to treatment failure of severe fungal infections (Nature Microbiology 2017).

The Mucorales order has been reported as prevalent in indoor environment and in occupational environments (Caetano, Faria, et al. 2017; Caetano, Zegre, et al. 2018; Viegas et al. 2018a). The resistance epidemiology of Mucorales in the environment remains to be fully elucidated. For this reason, the prevalence of Mucorales species indoor was determined in distinct occupational settings and dwellings, in independent projects. Here, we describe, for the first time to our knowledge, the combination of a culture-based method for a rapid azole-resistance screening in a broad range of environmental samples, with further identification of Mucorales isolates grown in azole-supplemented media by a Mucorales-specific real time PCR assay already tested in clinical samples, and their application to complex environmental samples.

## Materials and methods

### Projects for the assessment of Mucorales in the environment

Mucorales isolates were obtained from samples collected at independent projects aiming for the assessment of occupational exposure and indoor air quality, focusing on exposure to bioburden. Indoor environmental samples were collected between 2013 and 2018 from four different occupational settings around Lisbon metropolitan area, and from dwellings in Aveiro region (Portugal) (Table 1), as follows: bakeries (Caetano, Faria, et al. 2017; Caetano, Zegre, et al. 2018; Viegas et al. 2018c), swine farms (Viegas, Carolino, et al. 2013; Viegas, Faria, Dos Santos, et al. 2016b; Viegas, Faria, Monteiro, et al. 2018b), taxis used for patient transportation (Viegas et al. 2018d), waste-sorting plants (Viegas, Gomes, et al. 2014; Viegas, Faria, Dos Santos, et al. 2015; Viegas, Faria, Caetano, et al. 2017a), and dwellings (data not published). In order to collect and assess total bioburden and resistant mycobiota, different sampling devices were used per setting that were better adapted to the activities developed in each environment: electrostatic dust collector (EDC) from dwellings and bakeries; filters from the air-conditioning system of vehicles (taxis and waste fork lifters); litter and feed from swine farms; raw materials from bakeries (Table 1).

**Table 1.** Samples collected for Mucorales and total fungi assessment in each setting.

Project/ Setting	Area/Municipalities	Number of assessed units	Samples/ Matrices collected	Number of collected samples
Bakeries	Mafra Lisbon	10	EDC Raw material	27 26
Waste-sorting	Lisbon	2	Air-conditioning filter from fork lifter cabinet	17
Swine farms	Montijo Lisbon	5	Litter Feed	5 10
Taxis for patient transportation	Lisbon	19	Air-conditioning filter from taxi cabinet	19
Dwellings	Aveiro	79	EDC	79
**Total**		**115**		**183**

### Treatment of environmental samples

EDCs with a surface exposure area of 0.00942 m^2^ were placed at a minimum 0.93 m above floor level, and dust was allowed to settle for, at least, 15 days in bakeries and 30 days in dwellings. After sampling, EDCs were weighted and washed with 20 mL NaCl 0.9% with 0.05% Tween™ 80 by orbital shaking (250 rpm, 60 min) (Edmund Bühler SM-30, Hechingen, Germany) (Caetano et al. 2017; Viegas et al. 2018c).

Air-conditioning filters from waste fork lifters and from taxis used for patient transportation were removed from vehicles. All filters belonged to category 2 (≥3.0 µm pores) according to protection requirements (EN 15695), and were used for a maximum of 15,000 km in taxis and 22,240 working hours waste fork lifters. A piece of 2 cm^2^ was cut from each filter and kept refrigerated (4°C) before analysis. Filter pieces were washed with 10 mL of NaCl 0.9% with 0.1% Tween™ 80 (30 min, 250 rpm) on an orbital laboratory shaker, as previously described (Viegas, Faria, de Oliveira, et al. 2017b; Viegas, Monteiro, Dos Santos, et al. 2018d).

Litter (shredded journal paper) and feed (of non-specified cereal origin) from swine farms, and raw materials (including wheat, corn, malt, rye, barley, oats, malt, carob flours, and non-cereal ingredients such as flavourings and spices, baker’s yeast, sugar powder) from bakeries were collected, weighted and processed as previously described (Viegas, Carolino, et al. 2013; Caetano et al. 2017; Viegas, Faria, et al. 2018b). Briefly, 4.4 g of each (not oven-dried prior to processing, thus retaining natural water content) were washed with 40 mL of sterilised distilled water (20 min, 200 rpm) on an orbital shaker.

### Culture-based methods for fungal assessment

The fungal burden was determined through the inoculation of 150 µL of the wash suspensions on 2% malt extract agar (MEA) supplemented with 0.05% chloramphenicol and dichloran glycerol (DG18) agar supplemented with 0.01% chloramphenicol. DG18 was used due to its ability to restrict the colony size of fast-growing genera (Bergwall and Stehn 2002) allowing a more complete characterisation of fungal growth in complex matrices such as environmental and substrate samples. All the collected samples were also screened in azole-supplemented media by seeding 150 µL of the wash suspensions on Sabouraud agar media supplemented with 4 mg/L itraconazole, 1 mg/L voriconazole, or 0.5 mg/L posaconazole (adapted from the EUCAST 2017 guidelines) (EUCAST 2017). The inoculated plates were incubated at 27°C for 3–5 days, in order to allow the growth of all fungal species present in the samples. After the incubation period, fungal densities (calculated as colony-forming units (CFU) per 1 m^2^ of filter/EDC area, or CFU per 1 g of raw material/bedding/feed) were calculated. For species identification, microscopic mounts were performed using tease mount or Scotch tape mount and lactophenol cotton blue mount procedures. Morphological identification was achieved through macro and microscopic characteristics as noted by De Hoog *et al*. De Hoog (2000) by examiners with expertise in identifying fungi based on morphological and physiological characteristics.

### Mucorales-specific real-time PCR assay (Muc18S)

Mucorales-specific real-time PCR (qPCR) assay (Muc18S) was performed to achieve Mucorales identification to genus level, as previously described (Springer, Goldenberger, et al. 2016a; Springer, Lackner, et al. 2016b). Briefly, a locked nucleic acid probe was used to detect an approximately 175 bp amplicon. Clinically relevant Mucorales species such as *Cunninghamella sp., Lichtheimia sp., Mucor sp., Rhizomucor sp*. and *Rhizopus sp*. can be detected. For DNA extraction, 200 µl of spore suspension was used. Bead-beating cracked the spores using MagNA Lyser Green beads (Roche Diagnostics) and DNA was eluted by using a commercially available kit (High Pure PCR Template Preparation kit, Roche Diagnostics). Elution volume was adjusted to 70 µl (Viegas et al. 2018a). Amplicons were purified using the MinElute PCR purification kit (Qiagen) according to manufacturer’s instructions. The elution volume was 15 mL. Sequencing was done by a commercial company (LGC, Berlin, Germany). Sequences were identified through alignment with reference sequences using BLAST analysis (National Center of Biotechnology Information, Washington DC; www.ncbi.nlm.nih.gov/BLAST).

## Results

### Mucorales distribution

**Figure 1.**
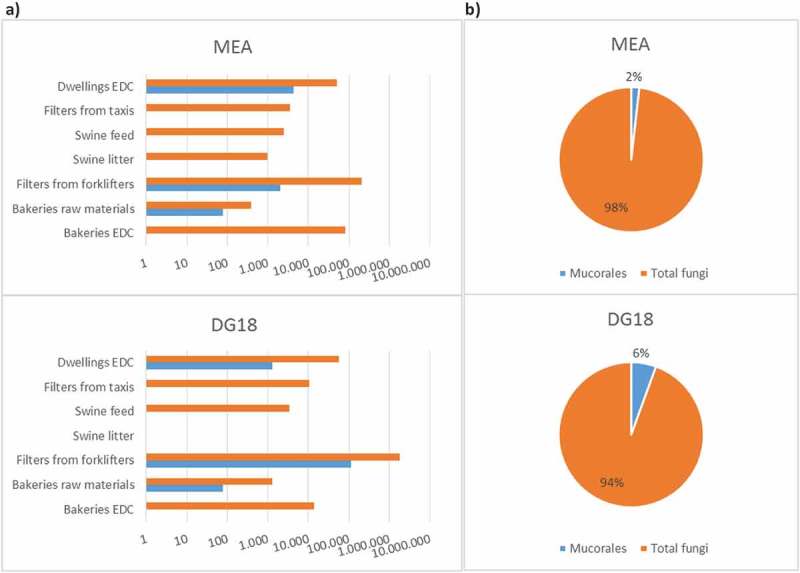
Mucorales and total fungi burden in malt extract agar (MEA) and in dichloran-glycerol agar (DG18) media: A) per sample type and per setting (log); B) Mucorales prevalence (%).

Mucorales burden in indoor samples collected by passive methods in occupational settings (*n* = 36) and in dwellings (*n* = 79) between 2013 and 2018 is shown in Figure 1. The total prevalence of Mucorales order in the assessed settings was 2% in MEA and 6% in DG18, as follows: 0% (MEA) to 1% (DG18) in bakeries; 1% (MEA) to 6% (DG18) in waste-sorting fork lifters; 0% (MEA and DG18) in swine farms and in taxis; and 2% (DG18) to 8% (MEA) in dwellings. There was substantial variation in the total fungal load and in the Mucorales load amongst the settings and, in some cases, amongst the different culture media. For example, samples collected in bakeries ranged from 0 to 76 CFU/m^2^ of EDC or CFU/g of raw material for both MEA and DG18, whereas samples collected in waste-sorting fork lifters ranged from 2000 CFU/m^2^ of filter in MEA to 110000 CFU/m^2^ in DG18, and samples collected in dwellings ranged from 1299 CFU/m^2^ of EDC in DG18 to 4299 CFU/m^2^ in MEA.

### Mucorales growth in azole-supplemented media

**Figure 2.**
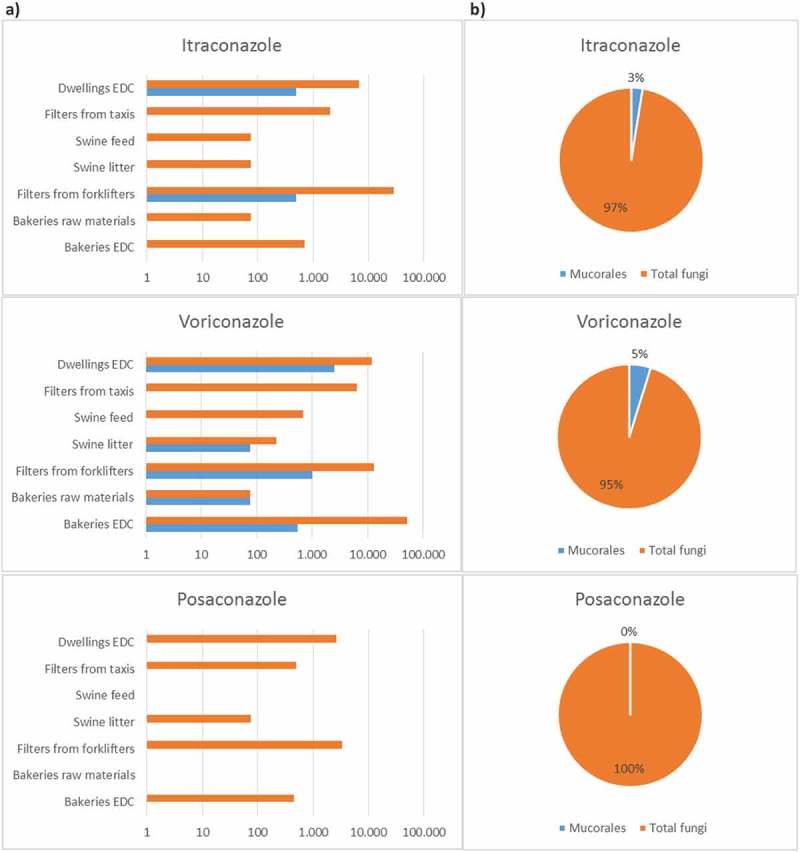
Mucorales and total fungi burden in azole-supplemented media: A) per sample type and per setting (log); B) Mucorales prevalence (%).

Figure 2 shows the distribution of Mucorales in environmental samples through growth in azole-supplemented Sabouraud media as a screening tool for resistance. Mucorales growth in at least one azole-supplemented media, except posaconazole, was observed in all settings in 14.8% (17 out of 115) of the collected samples, as follows: eight samples from dwellings; five samples from bakeries; two samples from waste-sorting fork lifters; one sample from swine farms and one sample from taxis. Mucorales growth was also observed in a second azole in waste-sorting industry and in dwellings (20 positive results). Mucorales growth in 1 mg/L voriconazole was observed in all settings, as follows: 76 CFU/g of raw material to 550 CFU/m^2^ of EDC in bakeries; 0 to 76 CFU/g of feed/litter in swine farms; 1000 CFU/m^2^ in waste-sorting fork lifters; 2498 CFU/m^2^ in dwellings; 1000 CFU/m^2^ in taxis. Mucorales load in 4 mg/L itraconazole was 500 CFU/m^2^ of filter in waste-sorting fork lifters, and 500 CFU/m^2^ of EDC in dwellings.

### Mucorales molecular detection and identification to genus level

Mucorales DNA was detected in all the isolates grown in azole-supplemented media (*n* = 20), and allowed the identification of Mucorales to genus level, corroborating the identification results obtained according to morphological criteria by culture-based methods. Amongst Mucorales isolates able to grow in azole-supplemented media, there was, as expected, a higher proportion of species less susceptible to 1mg/L voriconazole (16 out of 20 isolates, primarily *Mucor* sp., followed by *Rhizopus* sp. and *Rhizomucor* sp.) compared to the number of isolates less susceptible to 4 mg/L itraconazole (four isolates, primarily *Mucor* sp., followed by *Rhizopus* sp.) (Table 2).

**Table 2.** Molecular detection of Mucorales species distributed on azole-supplemented media.

Setting	Supplemented media	Muc18S/BLAST identification
Bakeries	VORI	Rhizopus
Bakeries	VORI	Mucor
Bakeries	ITRA	Mucor
Bakeries	VORI	Mucor
Bakeries	VORI	Mucor
Waste-sorting	ITRA	Mucor
Waste-sorting	VORI	Mucor
Waste-sorting	ITRA	Mucor
Taxis	VORI	Rhizomucor
Dwellings	ITRA	Rhizomucor
Dwellings	VORI	Rhizopus
Dwellings	VORI	Mucor
Dwellings	VORI	Rhizopus
Dwellings	VORI	Mucor
Dwellings	VORI	Mucor
Dwellings	VORI	Rhizopus
Dwellings	VORI	Rhizopus
Dwellings	VORI	Rhizopus
Dwellings	VORI	Mucor
Swine farms	VORI	Mucor

## Discussion

Mucormycosis is an emerging disease with limited treatment options. Limited therapeutic options for antifungal-resistant fungi that may evolve in the environment and display cross-resistance to drugs are an important public health threat to be addressed. Both the occupational and the living environments can be a source of azole-resistant mycobiota that, depending of the occupant’s health status, can be a serious public health problem (Lavergne et al. 2017). Thus, environmental, besides the clinical surveillance of azole resistance, should be considered to evaluate rates of azole resistance in each region/country (Lavergne et al. 2017).

Fungal exposure can be increased in confined environments, namely, in vehicles cabinets (such as waste sorting forklifters and taxis), in settings where high levels of particulate matter is generated and acts as fungi carrier, prompting higher levels of fungal dispersion (such as swine farms and bakeries), and also in buildings where ventilation or dampness might foster fungal colonisation (such as dwellings). Passive sampling is a versatile technique to detect bioburden in many settings allowing and improved characterisation of indoor environment. (Caetano et al. 2017; Viegas, Gomes, et al. 2014; Viegas, Faria, et al. 2016a; ).

Here, we describe the use passive sampling devices to assess Mucorales fungal burden and azole resistance in different settings based on their specific features (developed activities, work tasks, amount of hours spent indoor, occupants). The sampling strategy and methods for morphological and molecular characterisation of collected samples were based on the need to better acknowledge the presence of potentially harmful mycobiota indoor, including clinically relevant Mucorales species.

The use of passive methods in this study allowed the determination of fungal contamination levels indoor for both total and azole resistant mycobiota, and the specific identification of Mucorales, from a broad period of time (weeks to several months), whereas active methods (short-term air sampling) could only reflect the load from a shorter period of time (mostly minutes) with large spatial and temporal variations (Hyvärinen et al. 2001; Viegas et al. 2018c).

When possible, different sampling methods should be used in combination, to avoid having to rely in a single method and to represent a long-term time period–integrated scenario (Leppänen et al. 2018). This was the case in two (bakeries and swine farms) out of the five assessed settings, in order to obtain a more accurate risk characterisation (Viegas et al. 2018c) regarding resistant mycobiota in these settings. A wide spectrum mycobiota, with the identification of Mucorales order and of resistant mycobiota was, therefore, successfully achieved with this strategy.

The Mucorales load in MEA and in DG18 varied, overall as expected, in samples from the three settings where Mucorales was identified, with a lower load in DG18 in dwellings, and equivalent load in DG18 and in MEA in bakeries. One contradictory result was observed in samples from waste-sorting fork lifters, with Mucorales load 55-fold higher in DG18 than in MEA. The reason might be a restriction of other fungal species, and consequent equilibrium shift and increase of Mucorales (fast growing fungi) load in DG18 in relation to MEA. Owing to the lack of data regarding Mucorales specific assessment (Lackner et al. 2014) and the clinical importance of the Mucorales order (Kontoyiannis et al. 2005; Bitar et al. 2009; Auberger et al. 2012), the prevalence found in the three settings (including occupational environments and dwellings) should be of concern.

Ideally, and in light of the majority of results, for exposure assessments in occupational environments where high fungal contamination is present, such as waste-sorting industry, DG18 media should nevertheless be used to restrict fast growing fungi, such as Mucorales order, and the detection of Mucorales order in the collected environmental samples should be evaluated by molecular tools such as specific real time PCR assays, as applied in this study. Real-time assays can be run in a closed system, minimising contamination risk. As both quantification and identification are possible, monitoring of Mucorales DNA, e.g. in complex environmental samples, can provide contamination levels by genus, being a useful screening tool to guide prevention effectively in order to improve indoor air quality and minimise exposure to pathogenic fungi (Millon et al. 2013; Springer et al 2016a).

For a better understanding of the Mucorales burden, the concentration can be assessed by culture-based and molecular methods such as qPCR (Viegas et al. 2014). Of note, a similar approach has been suggested as a protocol for the assessment of *Aspergillus* in different occupational environments (Viegas et al. 2017a), with the application of culture-based methods coupled with molecular tools to allow a more refined, integrated and useful data. Like *Aspergillus* qPCR, the Mucorales qPCR assay used in our study provides high analytical specificity and consequent high degree of aetiological certainty at the genus level (Springer et al. 2016a). This approach is of added value for exposure assessments pursuing risk characterisation regarding fungal occupational exposure, as it enables: with culture-based methods, to determine Mucorales prevalence in each occupational and indoor environment whilst comparing quantitative information with guidelines; by applying the Muc18S assay for Mucorales DNA, to identify Mucorales genus without sequencing, thus, overcoming some constraints of culture-based methods (Lauriere et al. 2008), such as the underestimation of species belonging to Mucorales order if other fungal genera with also fast growing rates prevail, such as *Chrysonilia* sp. and *Trichoderma* sp.

The increased occurrence of opportunistic fungal infections in immunocompromised patients, and the emergence of antifungal resistance, both in the clinical and in the environment (Fairlamb et al. 2016; Nature Microbiology 2017) highlight the importance of addressing the prevalence of antifungal resistance and molecular detection of target species in the assessments of occupational exposure to fungal burden (Viegas et al. 2016b). In this study, a higher proportion of Mucorales isolates (16 out of 20, primarily *Mucor* sp., followed by *Rhizopus* sp. and *Rhizomucor* sp.) were, as expected, less susceptible to 1 mg/L voriconazole, and a lower, still significant, proportion of isolates (four out of 20, *Mucor* sp. and *Rhizopus* sp.) were less susceptible to 4 mg/L itraconazole. No Mucorales growth was observed in posaconazole. These results are in accordance with literature.

Of the azoles with significant anti-Mucorales activity, posaconazole and isavuconazole are effective and currently used for the treatment of mucormycosis (Dannaoui et al. 2003). Voriconazole lacks activity against Mucorales *in vitro* (Sun et al. 2002; Dannaoui et al. 2003; Imhof et al. 2004; Almyroudis et al. 2007; Vitale et al. 2012), with reports of breakthrough mucormycosis in patients under voriconazole prophylaxis confirming its limited efficacy (Imhof et al. 2004). Itraconazole exhibits species-specific *in-vitro* activity (Dannaoui et al. 2003; Vitale et al. 2012; Chowdhary et al. 2014; Espinel-Ingroff et al. 2015), with lower MICs for *Rhizomucor* sp. than for *Rhizopus* sp. and *Mucor* sp. Although a certain degree of *in-vivo* efficacy has been reported in animal models (Dannaoui 2017), itraconazole is not used in the treatment of patients with mucormycosis.

The fact that waste-sorting fork lifters and dwellings exhibited the higher contamination levels of Mucorales, both in non-supplemented and in azole-supplemented media, was surprising and is of concern, especially in dwellings, because they are inhabited by a wide range population, from children to elderly, as well as individuals with immunodeficiency or other disorders, thus, being at an increased risk of infection by opportunistic fungi (Hyvärinen et al. 2001; Nature Microbiology 2017; Lavergne et al. 2017; Leppänen et al. 2018). In occupational and indoor environments with high environmental prevalence of Mucorales order and azole-resistant strains, preventive and protective hygienic measures should be guided by such results.

In summary, these findings alert for an increased awareness for the necessary surveillance of Mucorales and azole resistance in the environment. More environmental assessments are necessary to provide local epidemiologic data if prevention measures are to be implemented on a sound basis. Risk characterisation of exposure to Mucorales is highly desirable, as mucormycosis is rapidly progressive, and, thus, adequate prevention measures and punctual antifungal therapy will substantially improve patient management. Molecular tools, and especially DNA-based detection by qPCR, may serve as a solid complimentary tool to culture-based methods for a more refined detection of environmental relevant isolates, often non-cultivable pathogens, in complex environmental matrices.

The higher prevalence of Mucorales found in MEA/DG18 and azole-supplemented media (including itraconazole) in waste sorting industry and dwellings suggest that the molecular study of mutations associated with secondary resistance to azoles would be important for a better characterisation of exposure to azole-resistant strains in high load settings. As such, future exposure assessments should comprise the following stages:

1. Culture-based methods to determine fungal load and Mucorales order prevalence in air and passive samples;
2. Targeting Mucorales order at genus level by refined molecular tools such as Muc18S assay in air and passive samples;
3. Screening of Mucorales growth in azole-supplemented media, namely, itraconazole, voriconazole and posaconazole;
4. Molecular identification of mutations related with secondary resistance in isolates of Mucorales grown in azole-media.

## Conclusions

This study describes the evaluation in different indoor environments of Mucorales prevalence and ability to grow in azoles using passive samples only and a combination of simple and fast culture-based and molecular tools. Considering the clinical relevance of Mucorales order and the obtained results, prevalence and azole-resistance surveillance should be ensured in different occupational and indoor environments, besides clinical facilities. Culture-based methods with supplemented media should be applied followed by a more refined molecular tool, such as Muc18S assay, for the detection of Mucorales species.
